# Autophagy Gene Variant *IRGM* −261T Contributes to Protection from Tuberculosis Caused by *Mycobacterium tuberculosis* but Not by *M. africanum* Strains

**DOI:** 10.1371/journal.ppat.1000577

**Published:** 2009-09-11

**Authors:** Christopher D. Intemann, Thorsten Thye, Stefan Niemann, Edmund N. L. Browne, Margaret Amanua Chinbuah, Anthony Enimil, John Gyapong, Ivy Osei, Ellis Owusu-Dabo, Susanne Helm, Sabine Rüsch-Gerdes, Rolf D. Horstmann, Christian G. Meyer

**Affiliations:** 1 Department of Molecular Medicine, Bernhard Nocht Institute for Tropical Medicine, Hamburg, Germany; 2 Institute of Medical Biometry and Statistics, University Hospital Schleswig-Holstein, Campus Lübeck, Lübeck, Germany; 3 National Reference Center for Mycobacteria, Research Center Borstel, Borstel, Germany; 4 Department of Community Health, College of Health Sciences, Kwame Nkrumah University of Science and Technology, Kumasi, Ghana; 5 Health Research Unit, Ghana Health Service, Accra, Ghana; 6 Komfo Anokye Teaching Hospital, Kumasi, Ghana; 7 Kumasi Centre for Collaborative Research in Tropical Medicine, Kumasi, Ghana; 8 Department of Parasitology, Bernhard Nocht Institute for Tropical Medicine, Hamburg, Germany; Johns Hopkins School of Medicine, United States of America

## Abstract

The human immunity-related GTPase M (IRGM) has been shown to be critically involved in regulating autophagy as a means of disposing cytosolic cellular structures and of reducing the growth of intracellular pathogens *in vitro*. This includes *Mycobacterium tuberculosis*, which is in agreement with findings indicating that *M. tuberculosis* translocates from the phagolysosome into the cytosol of infected cells, where it becomes exposed to autophagy. To test whether IRGM plays a role in human infection, we studied *IRGM* gene variants in 2010 patients with pulmonary tuberculosis (TB) and 2346 unaffected controls. Mycobacterial clades were classified by spoligotyping, IS*6110* fingerprinting and genotyping of the *pks1/15* deletion. The *IRGM* genotype −261TT was negatively associated with TB caused by *M. tuberculosis* (OR 0.66, CI 0.52–0.84, P_nominal_ 0.0009, P_corrected_ 0.0045) and not with TB caused by *M. africanum* or *M. bovis* (OR 0.95, CI 0.70–1.30. P 0.8). Further stratification for mycobacterial clades revealed that the protective effect applied only to *M. tuberculosis* strains with a damaged *pks1/15* gene which is characteristic for the Euro-American (EUAM) subgroup of *M. tuberculosis* (OR 0.63, CI 0.49–0.81, P_nominal_ 0.0004, P_corrected_ 0.0019). Our results, including those of luciferase reporter gene assays with the *IRGM* variants −261C and −261T, suggest a role for IRGM and autophagy in protection of humans against natural infection with *M. tuberculosis* EUAM clades. Moreover, they support *in vitro* findings indicating that TB lineages capable of producing a distinct mycobacterial phenolic glycolipid that occurs exclusively in strains with an intact *pks1/15* gene inhibit innate immune responses in which IRGM contributes to the control of autophagy. Finally, they raise the possibility that the increased frequency of the *IRGM* −261TT genotype may have contributed to the establishment of *M. africanum* as a pathogen in the West African population.

## Author Summary

Autophagy is a process in which cell components are degraded by the lysosomal machinery. It has recently been described that activation of autophagy reduces the viability of *M. tuberculosis* in phagosomes due to an intimate autophagy-phagocytosis interaction. *M. tuberculosis* may also be directly accessible to autophagy, as *M. tuberculosis* was found to translocate into the cytoplasm. The immunity-related GTPase IRGM is a mediator of innate immune responses and induces autophagy. We have studied genetic variants of the human *IRGM* gene in a Ghanaian tuberculosis case-control group and found that the *IRGM* variant −261T provides relative protection against disease when the infection is caused by the Euro-American lineage of *M. tuberculosis*. This lineage is characterized by the *pks1/15* seven base-pair (bp) deletion. The product of an intact *pks1/15* gene, phenolic glycolipid-tb, might contribute to mycobacterial virulence by suppressing innate immune responses. It is, therefore, conceivable that only the Euro-American lineage is exposed to IRGM-triggered innate defence mechanisms. Our observations suggest that the increased frequency of the *IRGM* −261TT genotype may have allowed the establishment of *M. africanum* as a pathogen in West Africa.

## Introduction

Autophagy is induced by the formation of intracellular double-membrane structures which form autophagosomes to sequester and, after further maturation to autolysosomes, degrade cytosolic protein aggregates and corrupted cellular organelles. Thereby, it allows a re-cycling of amino acids, which is of particular importance during periods of cell starvation. Autophagy is also an efficient innate defense mechanism in that it may lead to deposition of intracellular pathogens into an autophagosome for subsequent autolysosomal acidification and peptidase-mediated degradation. The net effect of well functioning autophagy is protein degradation, optimized loading of Human Leukocyte Antigen (HLA) class II molecules and intensified antigen presentation [1].

The activation pathways of autophagy and phagocytosis are closely related in that they share phosphatidylinositol-3-phosphate (PI3P) and other factors in facilitating the fusion step and closure of the phagophore at the final stage of phagosome and autophagosome formation [2]. Among other factors, immunity-related GTPases exert significant effects on both autophagy and phagosome maturation [3]. It was recently shown in human U937 cells that inhibition of the immunity-related GTPase IRGM by siRNA caused impaired conversion of light chain 3 (LC3), an exclusive marker of the autophagy cascade, into its active form which is required for the elongation of the double membranes and eventual completion of the autophagosome [4]. In these experiments, impaired LC3 conversion resulted also in an extended survival of BCG in phagosomes. These experiments were based on earlier experiments in mice where Irgm1 (syn.: LRG-47; encoded by Irgm1), the murine homologue of IRGM, was critically involved through induction of autophagy in the control of several intracellular pathogens, including *Mycobacterium bovis* BCG and *M. tuberculosis* H37rv [1],[3],[5],[6]. Notably, phagosome development into phagolysosomes may alternatively be induced by the autophagic component LC3 even when the conventional PI3P dependent pathway of phagosome maturation is blocked by experimental infection with the *M. tuberculosis* H37rv strain [3].

**Figure 1:**
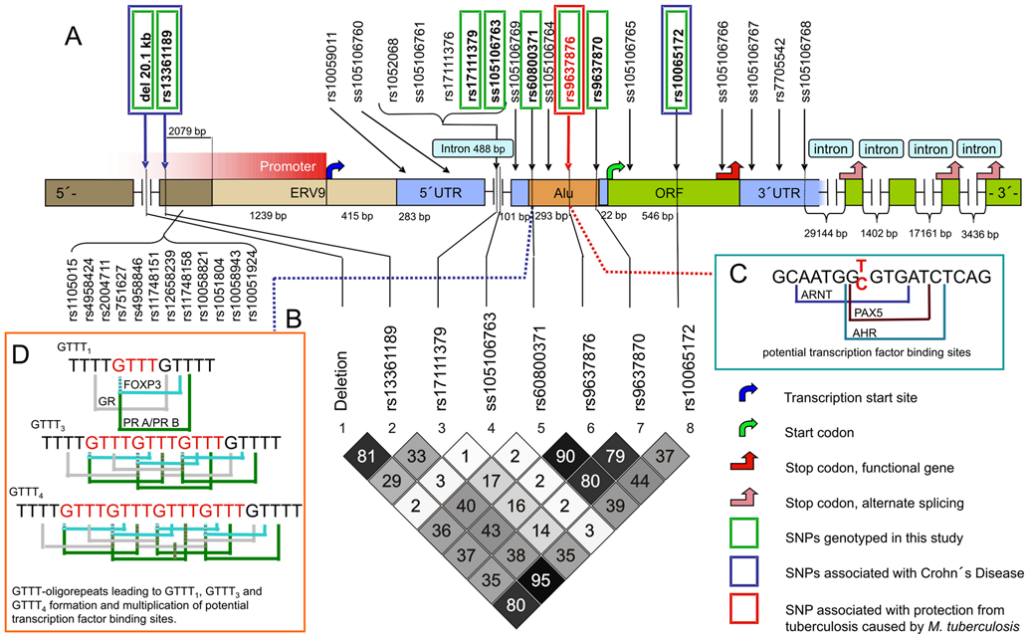
Schematic structure of the *IRGM* gene. A) Gene segments with lengths and all SNPs that are recognized so far are given. SNPs associated with Crohn's Disease, SNPs genotyped in the present study and the SNP associated with protection from TB caused by the EUAM-lineage of *M. tuberculosis* are indicated. The nucleotide positions of SNPs indicated by rs/ss numbers are listed in Table 1. B) Estimates of pairwise linkage disequilibria (LD) between *IRGM* variants with the LD measure of *r* ^2^ assuming Hardy-Weinberg equilibrium. C) Potential loss of transcription factor binding sites (ARNT, PAX5, AHR) in sequences carrying the *IRGM* −261T allele. D) Potential accumulation of transcription factor binding sites (FOXP3, GR, PR A/PR B), depending on the number of tetranucleotide repeats (rs60800371; [15]).

IRGM (syn: LRG47, IFI1) is encoded by the immunity-related GTPase protein family, M gene (*IRGM*; 5q33.1; OMIM *608212). *IRGM* consists of a long first exon encoding 181 amino acids, and four shorter putative exons that span more than 50 kilobases (kb) downstream of the first exon [7]. Most recent evidence indicates that the *IRGM* gene was deactivated during evolution but has regained its function after the insertion of a retroviral ERV9 segment and an Alu repeat sequence (Figure 1; [8]). Genetic variants of *IRGM*, including a 20.1 kilo-base (kb) upstream deletion polymorphism, have been found to confer in Caucasians an increased risk of developing Crohn's disease, whereby coding-sequence variation has been excluded to be the liable source of the association [9]–[11].

The phylogenetic tree of the *M. tuberculosis* complex contains two independent clades. Clade 1 represents all *M. tuberculosis sensu strictu* lineages that may be exclusively pathogenic for humans, while clade 2 comprises lineages that are pathogenic for both humans and animals (*M. africanum*, *M. bovis*) [12]. A major branch of *M. tuberculosis sensu strictu* is the *M. tuberculosis* Euro-American (EUAM) lineage that recently was shown to be prevalent and cause significant numbers of infections in West Africa as well [13]. While *M. tuberculosis* lineages occur throughout the world, *M. africanum* strains are almost exclusively restricted to West Africa [12],[14], suggesting the existence of factors favoring spread and preservation of *M. africanum* in West Africa.

Based on the *in vitro* evidence on the role of IRGM and autophagy in experimental *M. tuberculosis* infections of murine and human cells [3],[4] we hypothesized that naturally occurring *IRGM* variants, including the large upstream 20.1 kb deletion, might also be relevant to the phenotype of *in vivo* human pulmonary tuberculosis (TB). We have, in a case-control design, re-sequenced fragments of the *IRGM* gene and genotyped distinct genetic variants. An influence exerted by these variants on susceptibility or resistance to TB should be reflected in a large sample of active sputum-positive cases with pulmonary TB that we collected in Ghana, West Africa, and compared it to a control group of notable size.

## Results

### Power of the association study, Hardy-Weinberg equilibrium

A power of >90% of detection was achieved for multiplicative and additive models, assuming an approximative TB prevalence of 0.004 in West Africa, a frequency of 0.1 for high risk alleles, and a genotype relative risk of 1.3 (α = 0.05) with our sample size (case-control ratio = 1.18).

All variants tested were in HWE in cases and controls except *IRGM* −261 and *IRGM* −71, where the distribution of alleles was in HWE among cases, but deviated in controls. The deviation could be traced to the subgroup of controls of Ewe ethnic background. As *IRGM* −261 and *IRGM* −71 are in strong LD but were determined by independent genotyping assays, a genotyping failure appears to be highly unlikely. The most plausible explanation is that the deviation results from the low number of Ewe controls (5%). Calculational exclusion of this subgroup from statistical analyses did not affect the significance of our results.

As data on the frequency of the *IRGM* −261T variant in other ethnic groups are not available so far, we have typed this variant also in a small panel of 47 healthy Caucasian subjects. The genotypes CC, CT and TT were observed at frequencies of 0.81, 0.15 and 0.04, respectively, versus frequencies of 0.44, 0.43 and 0.13 in the Ghanaian study population. Although not representative due to the low number of Caucasian individuals and to the bias imposed by a non-randomly selected African study group, the data suggests that the *IRGM* −261T allele occurs by far more frequently among West Africans.

### Novel IRGM variants and genotypes identified by re-sequencing

Re-sequencing revealed, in addition to previously recognized polymorphisms, ten yet unidentified *IRGM* variants which were submitted to the NCBI dbSNP database (Table 1). The positions and preliminary NCBI ss numbers of the novel variants are as follows: *IRGM* −908A>C (ss105106760), −797C>T (ss105106761), −420C>T (ss105106763), −284G>A (ss105106764), 281C>A (ss105106765), 370A>G (ss105106766), *IRGM* intron +2T>C (ss105106767), intron +106T>C (ss105106768) and a deletion of two bases at positions −386/−387delAG (ss105106769). A tetranucleotide polymorphism (rs60800371; repeats starting at nucleotide position −308) was newly recognized to appear as triple repeat. This variant is located at the 5′-end of the Alu segment (Figure 1; [15]). Potential accumulation of transcription factor binding sites (FOXP3, GR, PR A/PR B) may occur, depending on the number of tetranucleotide repeats. All novel variants were confirmed by forward and reverse DNA sequencing and were observed in at least two different DNA samples. The *IRGM* genotypes identified by re-sequencing of 69 DNA samples are given in Table 1.

**Table 1:** *IRGM* variants and minor allele frequencies identified by re-sequencing.

Localization		rs-/ss number	Substitution	MAF
5′-UTR	−964	rs10059011	A>C	0.46 (A)
5′-UTR	−908*	ss105106760	A>C	0.01 (C)
intron	−844	rs10052068	T>C	0.23 (C)
intron	−797*	ss105106761	C>T	0.06 (T)
intron	−787	rs17111376	T>C	0.5
intron	−566	rs17111379	G>C	0.21 (C)
intron	−420*	ss105106763	C>T	0.06 (T)
5′-UTR	−386/387*	ss105106769	AG>del	0.04 (del)
Alu sequence	−308^#^*	rs60800371	1, 3 or 4 ‘GTTT’	
Alu sequence	−284*	ss105106764	G>A	0.04 (A)
Alu sequence	−261	rs9637876	C>T	0.31 (T)
Alu sequence	−71	rs9637870	G>A	0.31 (A)
exon 1	281*	ss105106765	C>A	0.01 (C)
exon 1	313	rs10065172	C>T	0.49 (T)
exon 1	370*	ss105106766	A>G	0.01 (G)
Intron	+2*	ss105106767	T>C	0.01 (C)
Intron	+87	rs7705542	G>A	0.49 (G)
Intron	+106*	ss105106768	T>C	0.01 (C)

Positions refer to the reference sequence and are given relative to the ATG start codon (reference sequence: chromosome 5 contig, GenBank accession number NW_922784.1, region 23935681…23938058; contains at position −308 one ‘GTTT’ motif). MAF, minor allele frequency; *, novel variant identified in the present study population; ^#^, microsatellite repeat of 1, 3 or 4 ‘GTTT’ tandem motifs.

### Mycobacterial genotypes

Out of the total of 1567 isolates that were obtained and characterized, 1029 (65.7%) were *M. tuberculosis* Euro-American (EUAM) strains (*pks1/15* 7 base-pair [bp] deletion), 472 (30.1%) were *M. africanum*, and 10 (0.6%) were *M. bovis* (the latter two lineages exhibiting the RD9 deletion). Fifty-six (3.6%) isolates belonged to the *M. tuberculosis* East-African-Indian (EAI), Beijing or Delhi lineages.

### Allele, genotype and haplotype associations

Genotyping of eight *IRGM* variants was performed in 2010 HIV-negative TB cases and 2346 controls. The genotyping detection rate was >94% for all variants.

In the entire study group, no allelic or genotypic association with disease or resistance was observed. A trend for an association with resistance to TB was, however, seen for the distribution of the *IRGM* −261TT genotype (OR 0.79, CI 0.65–0.96, uncorrected nominal P value [P_nom_] 0.017; Table 2). Stratification for the two major phylogenetic mycobacterial clades, *M. tuberculosis sensu strictu* and *M. africanum/M. bovis*, revealed a significant difference in the distribution of the *IRGM* −261 genotype among cases and controls. *IRGM* −261TT was significantly associated with protection from TB caused by *M. tuberculosis*, but not by *M. africanum/M. bovis* (OR 0.66, CI 0.52–0.84, P_nom_ 0.0009, P_corr_ 0.0045 vs. OR 0.95, CI 0.70–1.30, P_nom_ 0.8). The association was confirmed in additive and recessive statistical models (OR_add_ 0.86, CI 0.77–0.95, P_corr_ 0.025 and OR_rec_ 0.68, CI 0.53–0.85, P_corr_ 0.005, respectively; Table 3). Further stratification with respect to mycobacterial genotypes revealed that the association of the −261TT genotype applied exclusively to carriers of the *M. tuberculosis* Euro-American (EUAM) genotype, but not to individuals infected with *M. tuberculosis* East-African-Indian (EAI), Beijing or Delhi genotypes (OR 0.63, CI 0.49–0.81, P_nom_ 0.0004, P_corr_ 0.0019 vs. OR 1.20, CI 0.57–2.52, P_nom_ 0.6). Again, the association was substantiated in the additive and recessive models (OR_add_ 0.85, CI 0.76–0.95, P_corr_ 0.016 and OR_rec_ 0.64, CI 0.50–0.82, P_corr_ 0.0017, respectively; Table 4). No association was observed for carriers of the heterozygous *IRGM* −261CT genotype (OR 0.96, CI 0.82–1.13, P 0.6).

**Table 2:** *IRGM* genotype associations.

	GT	Cases (%)	Controls (%)	OR	CI	P_nom_	P_corr_	OR_add_	CI	P_nom_	P_corr_	OR_rec_	CI	P_nom_	P_corr_
***All genotypes***															
deletion	^WT^/_WT_	484 (24.7)	577 (25.2)	1				0.97	[0.89–1.05]	0.4		0.90	[0.78–1.04]	0.2	
	^WT^/_DEL_	1009 (51.5)	1131 (49.4)	1.05	[0.90–1.22]	0.524									
	^DEL^/_DEL_	466 (23.8)	584 (25.5)	0.93	[0.78–1.11]	0.430									
−4299	CC	545 (27.8)	677 (29.9)	1				1.05	[0.97–1.15]	0.2		1.03	[0.89–1.19]	0.7	
	CT	989 (50.4)	1104 (48.8)	1.11	[0.97–1.28]	0.14									
	TT	429 (21.9)	484 (21.4)	1.10	[0.93–1.31]	0.3									
−566	CC	117 (6.0)	118 (5.2)	1				0.99	[0.89–1.09]	0.8		1.01	[0.89–1.14]	0.8	
	CG	703 (36.1)	855 (37.3)	0.84	[0.64–1.11]	0.2									
	GG	1125 (57.8)	1318 (57.5)	0.87	[0.66–1.14]	0.3									
−420	CC	1787 (90.5)	2109 (91.6)	1				1.18	[0.96–1.46]	0.12		nc			
	CT	186 (9.4)	193 (8.4)	1.16	[0.94–1.43]	0.18									
	TT	2 (0.1)	0 (0.0)	nc											
repeat*	1/1	353 (18.2)	411 (18.1)	1				0.97	[0.94–1.01]	0.1		0.96	[0.93–1.00]	0.05#	
	1/3	386 (19.9)	416 (18.4)	1.09	[0.89–1.33]	0.4									
	1/4	602 (31.0)	687 (30.3)	1.03	[0.86–1.23]	0.8									
	3/3	88 (4.5)	117 (5.2)	0.89	[0.65–1.22]	0.5									
	3/4	272 (14.0)	305 (13.5)	1.04	[0.84–1.29]	0.7									
	4/4	243 (12.5)	331 (14.6)	0.86	[0.69–1.07]	0.17									
−261	CC	901 (45.2)	1011 (43.5)	1				0.91	[0.84–0.99]	**0.04**		0.80	[0.67–0.96]	**0.015**	
	CT	862 (43.3)	988 (42.5)	0.98	[0.86–1.11]	0.7									
	TT	229 (11.5)	324 (14.0)	0.79	[0.65–0.96]	**0.017**									
−71	GG	372 (39.0)	856 (38.9)	1				0.97	[0.89–1.06]	0.5		0.85	[0.71–1.01]	0.07	
	AG	465 (48.7)	999 (45.4)	1.08	[0.94–1.23]	0.290									
	AA	118 (12.4)	347 (15.8)	0.89	[0.73–1.07]	0.215									
313	TT	280 (27.9)	675 (29.2)	1				1.04	[0.95–1.13]	0.4		1.01	[0.87–1.17]	0.9	
	TC	506 (50.4)	1144 (49.5)	1.09	[0.94–1.25]	0.2									
	CC	218 (21.7)	491 (21.3)	1.07	[0.90–1.27]	0.5									

GT, genotype; OR, odds ratio; CI, 95% confidence interval; OR_add_, estimates of an additive genetic model. OR_rec_ estimates of a recessive genetic model; P_nom_ nominal P value; P_corr_, P value after Bonferroni correction (correction factor = 5); WT, wild-type, no deletion); DEL, 20.1 kb deletion; nc, not calculable.

*: number of tandem repeats given in digits.

# homozygous 4/4 genotype compared to all other combinations.

P values are adjusted for age, gender and ethnicity.

**Table 3:** *IRGM* −261 genotype associations after stratification for the two major groups of *M. tuberculosis* and *M. africanum*/*M.bovis*.

	GT	Cases (%)	Controls (%)	OR	CI	P_nom_	P_corr_	OR_add_	CI	P_nom_	P_corr_	OR_rec_	CI	P_nom_	P_corr_
***M. tuberculosis***															
−261	CC	502 (46.8)	1011 (43.5)	1				0.86	[0.77–0.95]	**0.005**	**0.025**	0.68	[0.53–0.85]	**0.001**	**0.005**
	CT	464 (43.3)	988 (42.5)	0.95	[0.82–1.11]	0.6									
	TT	106 (9.9)	324 (14.0)	0.66	[0.52–0.84]	**0.0009**	**0.0045**								
***M. africanum/M. bovis***															
−261	CC	204 (42.5)	1011 (43.5)	1				1.00	[0.87–1.15]	0.99		0.99	[0.89–1.10]	0.9	
	CT	214 (44.6)	988 (42.5)	1.07	[0.86–1.32]	0.5									
	TT	62 (12.9)	324 (14.0)	0.95	[0.70–1.30]	0.8									

GT, genotype; OR, odds ratio; CI, 95% confidence interval; OR_add_, estimates of an additive genetic model. OR_rec_ estimates of a recessive genetic model; P_nom_ nominal P value; P_corr_, P value after Bonferroni correction (correction factor = 5). P values are adjusted for age, gender and ethnicity.

**Table 4:** *IRGM* −261 genotype associations after stratification for strains positive and negative for the mycobacterial *pks1/15* deletion.

	GT	Cases (%)	Controls (%)	OR	CI	P_nom_	P_corr_	OR_add_	CI	P_nom_	P_corr_	OR_rec_	CI	P_nom_	P_corr_
***M. tuberculosis*** ** EUAM**															
−261	CC	476 (46.9)	1011 (43.5)	1				0.85	[0.76–0.95]	**0.003**	**0.016**	0.64	[0.50–0.82]	**0.0003**	**0.0017**
	CT	444 (43.7)	988 (42.5)	0.96	[0.82–1.13]	0.6									
	TT	96 (9.5)	324 (14.0)	0.63	[0.49–0.81]	**0.0004**	**0.0019**								
***M. tuberculosis*** ** EAI/Beijing/Delhi**															
−261	CC	26 (46.4)	1011 (43.5)	1				1.02	[0.70–1.49]	0.93		1.35	[0.67–2.70]	0.4	
	CT	20 (35.7)	988 (42.5)	0.78	[0.43–1.41]	0.4									
	TT	10 (17.9)	324 (14.0)	1.20	[0.57–2.52]	0.6									

GT, genotype; OR, odds ratio; CI, 95% confidence interval; OR_add_, estimates of an additive genetic model. OR_rec_ estimates of a recessive genetic model; P_nom_ nominal P value; P_corr_, P value after Bonferroni correction (correction factor = 5). EUAM, Euro-American lineages of *Mycobacterium tuberculosis*; EAI, East-African-Indian lineages of *Mycobacterium tuberculosis*. P values are adjusted for age, gender and ethnicity.

For the low numbers of patients infected with mycobacteria of the EAI/Bejing/Delhi group, the statistical power might not allow to obtain significant results and a possible association could be easily overlooked. To rule out this possibility statistically, a supplementary test of interaction was performed [16]. This test permitted to verify complete independence of ORs and validation of the exclusive liability of the *M. tuberculosis* EUAM genotype for the observed association. Cluster analyses of cases were done as previously described [17]. The distribution of the two variants at *IRGM* position −261 among and within clusters of cases did not differ significantly. This applied also when stratifications for ethnicity were performed.

Haplotypes and linkage disequilibria (LD) were reconstructed with the UNPHASED and Haploview softwares, respectively (Table 5, Figure 1). The corrected global P value of 0.017 for haplotypes after 1000 permutations comprising all variants that were tested indicated that the observed association might apply also to a distinct haplotypic combination. Detailed analyses including 1000 permutations for each combination showed that the *IRGM* haplotype 20kbdel_DEL/−4299C/−566G/−420C/rep4/−261T/−71A/313T was associated with resistance to TB caused by EUAM strains (OR 0.83, CI 0.73–0.93, P 0.001; Table 5), albeit the haplotype was not found to exert a stronger effect than the −261T variant alone when occurring homozygously (Tables 4 and 5). This underscores the significance of the *IRGM* −261TT genotype in protection from human pulmonary tuberculosis caused by the species *M. tuberculosis*.

**Table 5:** *IRGM* haplotype associations (8 polymorphisms).

Haplotype								Cases	Controls	OR	CI	P_nom_
Del	−4299	−566	−420	rep	−261	−71	313	n (%)	n (%)			
***M. tuberculosis*** ** EUAM**												
WT	C	G	C	3	C	G	T	28 (1.5)	65 (1.5)	1.06	[0.66–1.70]	0.8
WT	C	G	C	4	T	A	T	45 (2.3)	97 (21.9)	1.16	[0.80–1.69]	0.4
WT	T	C	C	1	C	A	C	24 (1.2)	50 (1.1)	0.98	[0.54–1.78]	0.95
WT	T	C	C	1	C	G	C	452 (23.5)	1009 (22.8)	1.05	[0.92–1.20]	0.4
WT	T	G	C	1	C	A	C	37 (1.9)	39 (0.9)	**1.93**	[1.14–3.27]	**0.01**
WT	T	G	C	1	C	G	C	340 (17.7)	784 (17.7)	1.00	[0.87–1.16]	0.96
WT	T	G	C	3	C	G	C	37 (1.9)	102 (2.3)	0.84	[0.57–1.23]	0.3
WT	T	G	C	4	C	G	C	28 (1.5)	52 (1.2)	1.25	[0.78–2.01]	0.4
DEL	C	G	C	3	C	A	T	25 (1.3)	37 (0.8)	1.38	[0.75–2.53]	0.3
DEL	C	G	C	3	C	G	T	264 (13.7)	553 (12.5)	1.10	[0.93–1.29]	0.3
DEL	C	G	C	4	T	A	T	554 (28.8)	1459 (32.9)	**0.83**	[0.73–0.93]	**0.001**
DEL	C	G	T	3	C	G	T	94 (4.9)	176 (3.9)	1.26	[0.97–1.63]	0.09

EUAM, Euro-American lineages of *Mycobacterium tuberculosis*; del, deletion; rep, repeat (number of tetranucleotide repeats given in digits; see also Figure 1); OR, odds ratio; CI, 95% confidence interval; P_nom_, nominal P value; WT, wild-type; DEL, 20.1 kb deletion. P values refer to comparisons of one haplotype to all other combinations and are adjusted for age, gender and ethnicity. Corrected global P value in cases caused by EUAM infections after 10000 permutations = 0.02. Missing genotypes are compensated for by simulations of all possible completions of the data.

### Reporter gene assay

**Figure 2:**
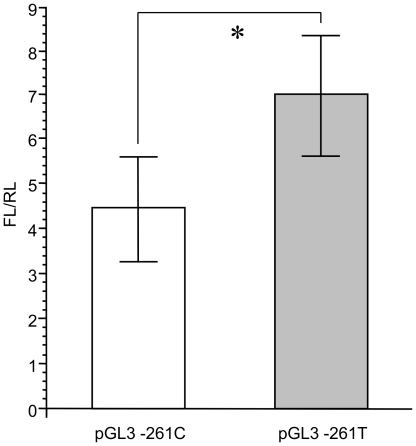
Comparison of pGL3 *IRGM* −261C and pGL3 *IRGM* −261T promoter constructs. FL/RL, ratio of firefly∶Renilla luciferase activity; pGL3 −261C, pGL3-Control Vector carrying the *IRGM* −261C wild-type variant; pGL3 −261T, pGL3-Control Vector carrying the *IRGM* −261T variant; *, P 0.013.

A two-tailed student's t-test revealed a significant difference in the normally distributed ratio of firefly∶Renilla luciferase activity of five independent transfections per *IRGM* variant in the luciferase reporter gene assay, indicating increased gene expression in cells transfected with pGL3-Control Vector carrying the mutant variant *IRGM*-261T than in cells transfected with the pGL3-Control Vector with the *IRGM* wild-type variant (−261C) (P = 0.013) (Figure 2).

## Discussion

We found in a large Ghanaian study group of HIV-negative patients with pulmonary TB and healthy control individuals that a distinct *IRGM* genotype, *IRGM* −261TT, was as a trend associated with decreased susceptibility to TB. After stratification for the two major mycobacterial clades and molecular subtypes the trend observed in the entire study group could be traced back to an association of *IRGM* −261TT with tuberculosis exclusively when caused by the *M. tuberculosis* EUAM lineage. No association was observed in comparisons of cases caused by *M. tuberculosis* EAI, Beijing, Delhi and *M. africanum/M. bovis* with controls. The frequencies of the synonymous variant 313T>C, the variant at position −4299 and of the 20.1 kb deletion, all found to be associated with Crohn's disease in other studies [9]–[11], did not differ significantly between TB patients and controls.

Upon infection of macrophages, *M. tuberculosis* initially resides in phagosomes. During their normal maturation, phagosomes fuse with lysosomes and establish a hostile environment for the pathogen, characterized by lysosomal enzymes, acid pH, reactive oxygen and nitrogen intermediates, and toxic peptides. Most macrophage mediated killing of intracellular pathogens occurs within the phagolysosome and pathogens have developed several mechanisms to avoid this vacuolar attack by escape and multiplication in the cytoplasm, inhibition of phagosome-lysosome fusion or circumventing the common endocytic pathway (reviewed in [18]). With regard to *M. tuberculosis* H37Rv, translocation of the bacteria from phagosomes to cytosolic compartments has been observed to occur in nonapoptotic cells [19]. Notably, escape into the cytosol has also been observed of the *M. tuberculosis* strain CDC1551 in laboratory infections of the amoeba *Dictyostelium discoideum* [20] and loss of phagosomal membranes with release of mycobacteria has been observed in an *in vitro* system five days post infection [21].

Autophagy induced by IRGM can efficiently interfere with cytosolic replication of pathogens by trapping and recapture and subsequent degradation of bacteria [22],[23]. In the LC3 dependent activation pathway, induction of autophagy contributes not only to the degradation of cytosolic components, but also to the maturation of mycobacterial phagosomes [3],[4]. While *M. tuberculosis* can impair phagosome maturation by blocking the normal PI3P dependent pathway, this effect is restored and outbalanced by the alternate activation mode triggered by LC3. This is consistent with the finding in mice and in human U937 cells that murine Irgm1 and human IRGM, respectively, play an important role in the containment of *M. tuberculosis* through efficient induction of autophagy [4].

The polymorphism that is associated with relative resistance to TB, *IRGM* −261T, in particular when occurring homozygously as TT genotype, might enhance expression of the mature IRGM protein which triggers autophagic degradation of translocated bacteria. The transcription factor AHR is expressed in the monocytic cell line THP1 that we used in our transfection experiments and inhibits differentiation of monocytes in vitro [24],[25]. This suggests that an unaffected AHR/ARNT transcription factor complex that is likely to occur in the *IRGM* wild-type gene promoter, may decrease innate immune responses mediated by macrophages. Inversely, and based on our luciferase reporter gene assays, the loss of the potential PAX5, AHR and ARNT transcription factor binding sites predicted for the *IRGM* variant −261T upstream of the coding region is likely to contribute to increased *IRGM* gene expression and, thus, enhanced innate responses. The fact that IRGM is not inducible by IFN-γ as are *Irg* genes in mice [4],[7],[26] but initiates successfully autophagy as does IFN-γ, argues for additional resistance mechanisms in individuals carrying the *IRGM* −261T variant homozygously, independent of other Th1-mediated immune responses.

The association that we observed was restricted to infections caused by the *M. tuberculosis* EUAM lineage. The major characteristic of that lineage is the *pks1/15* 7 bp deletion which does not occur in *M. africanum*, *M. bovis* and in the other *M. tuberculosis* lineages identified in our study, EAI, Beijing, and Dehli. Why relative protection by *IRGM* −261TT is exclusively provided against disease caused by the lineage that exhibits the *pks1/15* deletion remains to be explained further. One may hypothesize that absence or presence of the *pks1/15* deletion may contribute to a modulation of the pathogenic potential of infecting strains in different ethnicities, leading to an adaption of mycobacterial lineages to their sympatric host population. Inhibition of innate immune responses and a tendency of increased spread of bacteria by phenolic glycolipid TB (PGL-tb), the product of undamaged *pks1/15* gene, has been demonstrated [27],[28]. It is, therefore, reasonable to assume that lineages which do not produce PGL-tb as a suppressor of the innate immune response are more susceptible to IRGM-triggered innate immune mechanisms, namely phagosome maturation and autophagy. However, a more recent report indicates that PGL-tb itself does not confer hypervirulence, but rather differentially may modulate early cytokine response of the host [29]. As such, variable PGL-tb levels in conjunction with other yet inadequately defined mycobacterial virulence factors might result in a lineage-specific immunogenicity and/or degree of virulence that is related to the occurrence of human genetic variants in a particular population (14,29). Since translocation of bacteria from the phagosome has only been observed for *M. tuberculosis* H37rv and *M. leprae*, but not for *M. bovis* BCG [19], and was not tested for other lineages it is also conceivable that distinct pathogenic strains are subjected to mechanisms preventing translocation to cytosolic compartments and allowing escape from autophagy, a hypothesis that awaits further substantiation.

This would support the view of increased virulence of lineages carrying intact *pks1/15* genes such as *M. tuberculosis* Beijing and other lineages, but also underline the equivalent pathogenic potential of *M. africanum* compared to *M. tuberculosis* as has been observed in our study group [17]. It is intriguing to speculate that the high prevalence of *IRGM* −261TT in our Ghanaian study population and the relative protection that it confers from TB caused by *M. tuberculosis* EUAM might reflect one of multiple selection factors which promote the preferential and virtually exclusive propagation of *M. africanum* in West Africa, an assumption to be verified in other data sets of mycobacterial and human genotypes which are not yet available. The phylogeography of mycobacteria implies that lineages have become differentially adapted to different ethnicities with allelic variations conferring traits associated with certain infection phenotypes [14].

While the association of the *IRGM* polymorphism that we found cannot unquestionably confirm the role of IRGM in tuberculosis, it adds evidence to the in *vivo* experiments in mice and human cells [3],[4] and supports the relevance of autophagy in the control of tuberculosis. Unfortunately, genetic replication data on the role of human *IRGM* polymorphism in infections caused by *M. tuberculosis* subtypes that have unambiguously been determined by mycobacterial genotyping are not available so far, neither for Caucasian nor for African or Asian study groups. Data of functional experiments on the effect of IRGM in tuberculosis caused by strains that produce PGL-tb are not available. The inhibition of the innate immune mediators TNF-alpha and other interleukins by PGL-tb has been described earlier [26]. A corresponding inhibition of IRGM would explain our findings and contribute to understanding the role of PGL-tb as a factor of virulence and modulator of innate immune responses.

## Materials and Methods

### Ethics statement

The study protocol was approved by the Committee on Human Research, Publications and Ethics, School of Medical Sciences, Kwame Nkrumah University, Kumasi, and the Ethics Committee of the Ghana Health Service, Accra. Patients were treated according to the “Directly Observed Treatment, Short-course” (DOTS) strategy organized by the Ghanaian National Tuberculosis Programme. Blood samples for genetic analyses and HIV testing were taken only after a detailed explanation of the study aims and written or thumb-printed consent for participation provided, including HIV testing.

### Patients and controls

Study participants were recruited in Ghana, West Africa, between September 2001 and July 2004. The recruitment area and the enrollment procedure have previously been described [17],[30]. Patients were enrolled at the two Teaching Hospitals in Accra and Kumasi and at additional hospitals or policlinics in Accra, Tema, Kumasi, Obuasi, Agona, Mampong, Agogo, Konongo and Nkawie (Ashanti Region), Nkawkaw and Atibie (Eastern Region), and Assin Fosu and Dunkwa (Central Region). Characterization of patients included i) the documentation of the medical history on standardized structured questionnaires, including self-reported duration of cough and symptoms of TB (dyspnea, chest pain, night sweats, fever, hemoptysis, weight loss), ii) two independent examinations of non-induced sputum specimens for acid-fast bacilli, iii) serological determination of the HIV status and confirmation of positive results by an alternative test system, iv) culturing and molecular differentiation of phylogenetic mycobacterial lineages, and v) a posterior-anterior chest radiography. Inclusion criteria were two sputum smears positive for acid-fast bacilli, no history of previous TB or anti-mycobacterial treatment and an age between 6 and 60 years. Two sputum smears were examined in order to corroborate and confirm the phenotype. Patients were also included if only one smear and the culture for mycobacterial growth was positive. Exclusion criteria were incomplete information provided on the questionnaire, HIV positivity, evidence of alcoholism, drug addiction and other apparent generalized disease. A total of 2010 patients fulfilling the criteria for participation were enrolled.

Unrelated personal contacts of cases and community members from neighboring houses of cases and public assemblies were recruited as controls. The leading criterion for enrollment as a control was no history of TB or previous anti-mycobacterial treatment. Characterization of controls included a medical history, posterior-anterior chest X-ray radiography and a tuberculin skin test (Tuberculin Test PPD Mérieux, bioMérieux, Nürtingen, Germany). 1211 personal contacts and 1135 community members fulfilled the criteria for participation and were available as controls.

Study participants belonged to the following ethnic groups (cases/controls): Akan including Ashanti, Fante, Akuapem (63.6%/59.1%), Ga-Adangbe (14.5%/19.8%), Ewe (7.1%/9.3%) and ethnic groups of northern Ghana including Dagomba, Sissala, Gonja, and Kusasi (12.9%/10.4%). The proportions of ethnicities among patients and controls did not differ significantly.

Disclosure of HIV test results was dependent on the documented willingness of participants to be informed and included for HIV-positive patients their prompt referral to counseling and treatment provided by the Ghanaian AIDS Control Programme.

### Typing of mycobacterial isolates

The firm diagnosis of pulmonary TB was made as described previously [13],[17],[30]. *M. tuberculosis* complex isolates were cultured on Löwenstein-Jensen (LJ) media and shipped to the German National Reference Centre for Mycobacteria (Borstel, Germany) for minute analyses of biochemical, growth and molecular characteristics. Molecular differentiation of 1567 mycobacterial isolates included spoligotyping, IS*6110* fingerprinting and typing of the *pks1/15* deletion as described previously [31]–[33]. Mycobacterial strains were for further stratification grouped according to the major phylogenetic lineages [12].

The stepwise procedure of typing of mycobacteria included an initial cluster analysis of IS*6110* fingerprinting data and lineage identification according to specific spoligotype signatures. Assignment of lineages was based on the MIRU-VNTRplus webpage (www.miru-vntrplus.org; [34]) and a reference strain collection using the Bionumerics 5.1 software (Applied Maths, Sint-Martens-Latem, Belgium). Classification was confirmed by random selection of 20 strains of each group and testing for the presence of lineage-specific deletions as follows: *pks1/15* for *M. tuberculosis* Euro-American, region of difference (RD) 239 for *M. tuberculosis* East African Indian (EAI), RD9 and RD711 for *M. africanum* West African 1, RD9 and RD702 for *M. africanum* West African 2, and RD9 and RD4 for *M. bovis*. All *M. tuberculosis* strains with ambiguous lineage identification were confirmed as belonging to the Euro-American lineage by identifying the presence of the *pks1/15* 7 bp deletion. Deletion typing was performed using protocols available at the MIRU-VNTRplus webpage [34].

Clusters were defined as groups of affected individuals infected with mycobacterial strains exhibiting the same IS*6110* fingerprinting patterns. If less than 5 bands were identified, analyses were supplemented by spoligotyping. Identical strains isolated from 2 or more patients were regarded as a cluster and strains found in 1 patient only were considered unique.

### HIV testing

For HIV-1/-2 testing of TB cases, a capillary test system (Capillus, Trinity Biotech, Bray, Co Wicklow, Ireland) was applied. HIV positivity was confirmed by the Organon Teknika Vironostika HIV-1/-2 EIA system (Organon Teknika, Turnhout, Belgium). The rate of confirmation was 100%. HIV-positive TB patients were excluded from further genetic analyses.

### DNA re-sequencing of *IRGM*

DNA was isolated from peripheral blood samples of participants (AGOWA® mag Maxi DNA Isolation Kit, Macherey & Nagel, Germany) following the instructions of the manufacturer. The reference sequence was derived from the chromosome 5 contig, GenBank NW_922784.1, region 23935681…23938058. In order to reliably identify the *IRGM* variants occurring in our study population and to select variants for genotyping, a segment containing 1053 bp of the 5′UTR region upstream of the ATG start codon including the intron and the Alu segment, the open reading frame (ORF) of 546 coding bp and 250 bp of the 3′ region distal of the ORF of the *IRGM* gene were sequenced. Sequence analysis was performed in 23 TB patients, 23 PPD-positive and in 23 PPD-negative controls. Sequencing reactions were run on an automated ABI 3100 DNA sequencer (Applied Biosystems, Foster City, USA). Forward (F) and reverse (R) primers for re-sequencing were IRGM_pro1000F ccttgaaaaagagcagagcatt and IRGM_pro1000R tagcatccccagccctca (598 bp), IRGM_pro500F ttgctccctgaagaaatgtg and IRGM_pro500R ctcaacattcatggcttcca (599 bp), IRGM_p1F aatatctgcgtccagggttc and IRGM_p1R tgaactgcatttccatcagg (572 bp) and IRGM_p2F tgtgcctcctatttctcttcc and IRGM_p2 tgatataatcttgcatccattttaag (600 bp).

### *IRGM* variants selected for genotyping

Based on the results of re-sequencing and evidence of association of with other conditions, eight *IRGM* variants were selected for genotyping (Table 6). The variant at position −566G/C (rs17111379) was chosen for genotyping, as this variant was by re-sequencing identified in the groups of TB cases and PPD-positive controls only. Notably, *IRGM* −566 allele frequencies are unevenly distributed between Caucasians and the West African ethnicity of Yoruba (Caucasians: G 1.0; Yoruba, originating from Nigeria: G 0.74, C 0.26; www.hapmap.org/cgi-perl/snp_details?name=rs17111379&source=hapmap_B35). The variant at position −420C/T (ss105106763) was included as it was identified as a novel variant in our study population and was not in strong linkage with other polymorphisms. The *IRGM* variant −261 (rs9637876) was selected for genotyping, because, according to an *in silico* prediction of transcription factor binding properties, the allele causes loss of several binding sites (PAX5, ARNT, AHR; http://alggen.lsi.upc.es/recerca/menu_recerca.html). *IRGM* −71 (rs9637870) and the microsatellite repeat (rs60800371; repeats starting at position −308) are in LD with *IRGM* −261 and were included in genotyping to more reliably identify a potentially causative variant. The synonymous exonic variant 313C/T (L105L; rs10065172), the 20.1 kb deletion and the variant located 124 bp downstream of the 20.1 kb deletion (rs13361189; position −4299) were included in the genotyping panel due to their proven association with Crohn's disease [9]–[11].

**Table 6:** Primer Pairs and sensor/anchor oligonucleotides for LightTyper-based *IRGM* genotyping.

*IRGM*	Variant, rs #	Localization	Primer oligonucleotides	Sensor/Anchor oligonucleotides
20.1 kb deletion	WT/DEL na	upstream	F1-GCACTGGTGGACAGGTAA	S-Cy5-GGCTTTTTCTGTGACCATGTCTGTGAAC-phosphate
−24525–−4423			F2-CATTTACAGTAGTATCAAAAGAATAAAAACAATTTTATTTAAA	
			R-TGCTTGGGTACGACACG	A-6-Fam-TGTGGGAGGTCAGCAGAAAACCCTATAGTTTTCC
−4299	C/T, rs13361189	upstream	F-CCCACACTCACCATGGA	S-AATCGGATGTATATTAGTAGACCCCGTG-6-Fam
			R-ACATAGTACACTTTGTGTGTTG	A-Cy5-CAGCGGGTACAGTTTAGAAAGGGAAGTT-phosphate
−566	G/C, rs17111379	intron	F-CTGCTTTGGAGGCTGAC	S-Cy5-CTCCCTGAACAAATGTGCACATTG-phosphate
			R-AGGTTAAGGATGCAGCTAATAG	A- AGGGCGTTCCTCAGGGACCCTACCTTTCTT-6-Fam
−420	C/T, ss105106763	intron	F-CTGCTTTGGAGGCTGAC	S-TCACCTGCTCTTCTACTTCCGC-6-Fam
			R-AGGTTAAGGATGCAGCTAATAG	A-Cy5-GCTTACTCCAGTGCCCACAGATACGACAG-phosphate
repeat	1–4, rs60800371	Alu sequence	F-CATCCTAACTCACCTGCTCTTCTA	S-6-Fam-TAAGCATTGGGTTTTGTTTGTTTGTTTGTTTGTTTTGAGA
−308–−311/−319/−323			R-TGTTAGAGATCACCTCTGGCAA	A-Cy5-GGAGTCTTGCTCTGTTGCCAGGCTGGAG-phosphate
−261	C/T, rs9637876	Alu sequence	F-CCAGTGCCCACAGATAC	S-CTGGAGTGCAATGGCGTGAT-6-Fam
			R-CAGCTAATACAGGAGGCTGA	A-Cy5-TCAGCTCACTGCAATATCTGCGTCCAGGG-phosphate
−71	A/G, rs9637870	Alu sequence	FCACGCGCAGCTAATTTTTTTGTATTTTA	S-Cy5-CACTTTGGAAGGCCGAGGCGA-phosphate
			R-TTCAGAGTCTCCTTGATGTTA	A-6-Fam-CGCCGTGGCTCATGCCTGTAATCCC
313	C/T, rs10065172	Exon 1	F-CCTCACCTCCTACTGAGC	S-GAGAACTACCTGATGGAAATGCAGTTCA-6-Fam
			R-TTTCCCATGTCCTCAGCG	A-Cy5-CCGGTATGACTTCATCATGGTTGCATCTGCACA-phosphate

F, forward primer; R, reverse primer; F1, F2, two forward primers applied for the assessment of the 20.1 kb deletion; S, sensor oligonucleotide; A, anchor oligonucleotide; na, not available; 6–Fam, 6–carboxyfluorescein; Cy5, corresponding to the Roche fluorophore LC Red 640; synthesized by biomers.net GmbH, Ulm, Germany.

Further variants that were identified by DNA re-sequencing were not subjected to genotyping, as they were either in perfect LD with +313 (−964A/C, −787C/T, +87A/G), or too low in their frequencies to allow for reasonable statistical calculations (−908A/C, −844C/T, −797C/T, −386/7delAG, −284G/A, 281C/A, 370A/G, +2T/C, +106C/T).

### Genotyping of *IRGM* variants

*IRGM* variants were analyzed by dynamic allele-specific hybridization with fluorescence resonance energy transfer (FRET) in a LightTyper device (Roche Diagnostics, Mannheim, Germany). Primer pairs, sensor- and anchor-oligonucleotides utilized are listed in Table 6.

As no frequency information was available for the occurrence of the *IRGM* −261 polymorphism among Caucasians, this variant was also genotyped in 47 healthy voluntary German individuals (employees of the Bernhard Nocht Institute for Tropical Medicine, Hamburg; informed consent obtained).

For the microsatellite repeat, a FRET-based LightTyper method was developed using a sensor nucleotide that covered the entire tetranucleotide repeat with adjacent 5′- and 3′-nucleotides [15]. Depending on the actual number of the repeats, distinct melting temperatures allow explicit specification of the number of repeats. For the 20.1 kb deletion upstream of the *IRGM* promoter, the LightTyper design was combined with a gap-PCR comprising two forward primers matching upstream and within the region of deletion. The assay enabled the determination of gene fragments with and without the 20.1 kb deletion.

### Plasmid construction and reporter gene assay

All plasmid constructions were based on the pGL3-Control Vector (Promega, Mannheim, Germany) which contains the SV40 promoter driving the firefly luciferase gene. Two fragments, each of 0.4 kb, comprising the Alu-repeat sequence and, thus, including the *IRGM* variants of interest, −261C or −261T, were PCR-amplified with oligonucleotide primers 5′-cgaagctttcacactctattagctgcatccttaac-3′ and 5′-cgccatggctttctcaacattcatggcttccat-3′ from 10 ng of genomic human DNA (Biomers.net GmbH, Ulm Germany).

PCR conditions were: Initial denaturation (98°C, 30′), 30 amplification cycles (98°C, 7′; 64°C 20′; 72°C, 35′) and final elongation (72°C, 7′). *Hind*III and *Nco*I restriction sites at the 5′ and 3′ end, respectively, were engineered on each PCR product. Fragments were then ligated into the *Hind*III-*Nco*I digested pGL3-Control Vector to generate plasmids pGL3 −261C and pGL3 −261T. Enzymatic digestions and ligations were performed according to the instructions of the manufacturer. Small-scale preparations were done applying the NucleoSpin Plasmid Kit (Macherey and Nagel, Düren, Germany). Plasmid DNAs were propagated in *E. coli* XL1- Blue cells (Stratagene, La Jolla, CA, USA) and prepared using the EndoFree Plasmid Maxi Kit (Quiagen, Hilden, Germany). DNA sequences of the final constructs were confirmed by sequencing.

1×10^6^ cells of the human monocytic cell line THP1 (German Resource Centre for Biological Material, DMSZ, Braunschweig, Germany) were transfected with either 0.5 µg of the two plasmid constructs, with the addition of 0.5 µg of the plasmid phRL-CMV which contains the gene encoding Renilla luciferase (Promega, Mannheim, Germany) in order to normalize results. Transfectiosn with 0.5 µg of the unmodified pGL3-Control Vector and 0.5 µg phRL-CMV, as well as a mock transfection, were performed for control and to minimize effects of reagents on the cells. All transfections were performed with the Amaxa Cell Line Nucleofector Kit V (Lonza, Cologne, Germany). Four hours after transfection, cells were harvested and luciferase activities were measured (Dual-Luciferase Reporter Assay System; Promega, Mannheim, Germany). Firefly luminescence was measured using a single tube Junior LB9509 luminometer (Berthold Technologies, Bad Wildbad, Germany). After a 10 second measurement period, 100 µl 1× Stop & Glo Reagent were added for the detection of Renilla luminescence. Measurements of luminescence are expressed as relative light units (RLU). For each variant, five independent transfections were performed.

### Databases, statistics

Demographic data, self-reported signs and symptoms as documented on structured questionnaires as well as laboratory results were double-entered into a Fourth Dimension database (San Jose, CA, USA). Bacteriological data were provided as Excel datasheets. Data were locked before using them in a pseudonymized form for statistical analyses. Power calculations were performed with the public CATS software (http://www.sph.umich.edu/csg/abecasis/CaTS/).

Multivariate logistic regression analyses were calculated for different models to determine odds ratios (OR) for allele and genotype distributions (STATA 10.0MP software; Stata Corporation, College Station, TX, USA). As age, sex and ethnicity were significant confounders, they were appropriately adjusted for. Analyses of allele distributions and Hardy-Weinberg equilibria (HWE) were calculated with a public STATA module (www-gene.cimr.cam.ac.uk/clayton/software/stata/genassoc; David Clayton, Cambridge, UK). Haplotypes were estimated with the UNPHASED software (version 3.0.13; Frank Dudbridge, http://www.mrc-bsu.cam.ac.uk/personal/frank/software/unphased/), whereby incomplete haplotypes were subjected to simulations comprising all possible haplotypic completions of available data. In order to verify the independence of ORs that were obtained for associations of different mycobacterial clades/genotypes with phenotypes, ORs were subjected to tests of interaction according to the method described in [16].

Corrections of nominal P values (P_nom_) for multiple testing applied to the number of variants tested and when stratifications by mycobacterial lineages were made. Bonferroni corrected P values (P_corr_)<0.05 were considered significant and correction values are indicated where applicable.

The tetranucleotide repeat rs60800371 and SNPs at positions −261 (rs9637876) and −71 (rs9637870) are in strong LD (r^2^∼0.8) and were, therefore, combined as a single correction entity. Global P values of haplotype associations and of distinct haplotypes were subjected to 1000 permutations (UNPHASED software).

Shapiro-Wilk tests and a two-tailed student's t-test were calculated to confirm a normal distribution and differences in the ratio of firefly∶Renilla luciferase activity in the reporter gene assay.

### Accession numbers

#### Genes and genomic contig

Immunity-related GTPase family, M; *IRGM* (NCBI BC128168.1); Homo sapiens chromosome 5 genomic contig (NCBI NW_922784.1).

#### Protein

Immunity-related GTPase family, M; IRGM (NCBI NP_001139277).

#### Transcription factors

Aryl hydrocarbon receptor nuclear translocator; ARNT; (HUGO Gene Nomenclature Committee, [HGNC] HGNC:700); Aryl hydrocarbon receptor; AHR (HGNC HGNC:348); Paired box 5; PAX5 (HGNC HGNC:8619); Forkhead box P3; FOXP3 (HGNC HGNC:6106); Nuclear receptor subfamily 3, group C, member 1/glucocorticoid receptor; GR (HGNC HGNC:7978); Progesterone receptor/PGR; PR A/PR B (HGNC HGNC:8910).

#### Single nucleotide polymorphisms

Deletion (not available); −4299 C>T (NCBI rs13361189); −964 A>C (NCBI rs10059011); −908 A>C (NCBI ss105106760); −844 T>C (NCBI rs10052068); −797 C>T (NCBI ss105106761); −787 T>C (NCBI rs17111376); −566 G>C (NCBI rs17111379); −420 C>T (NCBI ss105106763); −386/387 AG>del (NCBI ss105106769); −308 1, 3 or 4 ‘GTTT’ (NCBI rs60800371); −284 G>A (NCBI ss105106764); −261 C>T (NCBI rs9637876); −71 G>A (NCBI rs9637870); 281 C>A (NCBI ss105106765); 313 C>T (NCBI rs10065172); 370 A>G (NCBI ss105106766); +2 T>C (NCBI ss105106767); +87 G>A (NCBI rs7705542); +106 T>C (NCBI ss105106768).
